# Cell-matrix interactions in dermal repair and scarring

**DOI:** 10.1186/1755-1536-3-4

**Published:** 2010-03-11

**Authors:** Beate Eckes, Roswitha Nischt, Thomas Krieg

**Affiliations:** 1 Department of Dermatology, University of Cologne, Cologne, Germany

## Abstract

Regulation of cellular functions during dermal repair following injury is complex and critically dependent on the interaction of cells with the surrounding extracellular matrix (ECM). The ECM comprises various families of macromolecules that form the structural scaffold of the tissue, but also carry distinct biological activities. After injury to the skin, the defect is filled by a provisional matrix that is invaded by inflammatory cells, sprouting blood vessels and fibroblasts. In a later phase, the wound contracts, the tissue is replaced by mature connective tissue produced by activated fibroblasts, and a scar is formed. All cells involved communicate directly with the ECM by integrins and other matrix receptors. These transmit signals and induce adaptive responses to the environment by the embedded cells. The ECM or proteolytic fragments of individual ECM constituents exert defined biological activities influencing cell survival, differentiation of myofibroblasts, ECM synthesis and turnover, wound angiogenesis and scar remodeling. Extensive crosstalk exists between ECM and growth factors, and between growth factors and integrins. ECM-cell contact also enables direct transmission of mechanical tension, which then modulates many activities of all cellular players. Understanding this complex interplay is important to provide a basis for designing effective wound therapy and for strategic interference with mechanisms that have gone out of control in fibrotic conditions.

## Review

The functioning of cells is critically dependent on their interaction with the surrounding extracellular matrix (ECM) [1]. These cells include not only fibroblasts, osteoblasts and chondrocytes but also endothelial, inflammatory, epithelial and smooth muscle cells. The direct interaction of these cells with the ECM is mediated by specific cellular receptors, which bind the ECM components, but they can also involve other cell surface glycoproteins that indirectly mediate the contact with structural components of the ECM. Cell-ECM interactions further enable the transmission of mechanical forces, which are generated in all tissues. The cellular activities controlled by this interaction are required for normal development, but they are also crucially involved in several physiological and pathological processes, in particular wound healing, scarring and fibrosis. In this review, the individual players orchestrating the cellular response after tissue injury to the skin are discussed.

## The extracellular matrix

Research over the past 20 years has increased our understanding of the composition of the ECM structures, and identified and characterized distinct families of matrix proteins (Appendix 1). These protein families include several subfamilies of collagenous proteins, adhesive glycoproteins, proteoglycans and matricellular proteins. A common denominator is their composition from a limited number of structural domains. It is important to note that these individual domains carry biological activities, modulate the activity of growth factors and cytokines, and harbor recognition sites for the interaction with specific cell membrane receptors [2,3]. ECM proteins form the structural scaffold of all tissues. They interact closely with each other, thereby constructing large networks with structural and non-structural components. In addition, they have many more specialized functions that regulate cellular behavior either by direct cell-ECM interaction or by the modulation of growth factor activities [4]. Synthesis, deposition and remodeling of ECM proteins are essential for the restoration of damaged tissue during the early and late phases of wound healing.

### Composition and function

#### Collagens

Collagens represent a group of diverse protein subfamilies containing at least 28 different collagen types that are encoded by more than 42 genes [3,5]. All members share a common structural feature, the presence of at least one triple helical domain. Their structural organization and supramolecular assembly depends on the specific subfamily of collagens, thus the interstitial collagens aggregate into fibrils, collagen VI aggregates into microfibrillar structures, collagen IV and VIII build large networks, and collagen VII forms the anchoring fibrils in the skin. Further subfamilies include fibril-associated collagens with interrupted triple helices, multiplexins (multiple triple helical domains and interruptions) and transmembrane collagens (Appendix 2). Multiplexins carry glycosaminoglycan chains and are thus considered not only as collagens, but also as proteoglycans. The transmembrane collagens contribute to the formation of cell-ECM contact sites such as hemidesmosomes and focal adhesions. They can be proteolytically shed from the cell surface, and the shed forms are detectable in the ECM and in body fluids. Processing is not unique to transmembrane collagens as many of the other large ECM molecules such as multiplexins are also proteolytically modified. Cleavage generates fragments that carry new biological activities, several of which regulate angiogenesis directly or indirectly, for example, by modulating integrin binding [6].

Endostatin, a fragment of collagen XVIII, is one of the best-characterized collagen fragments [7]. It is an inhibitor of angiogenesis and has been intensively investigated as an anti-angiogenic peptide in tumor models [8]. Findings about its role in dermal wound healing are controversial. The anti-angiogenic proteolytic fragments of collagen IV, tumstatin and canstatin have been also studied in wound healing, but no effect was noted [9]. For the more recently identified collagen XV fragment, restin, no wound healing data are yet available [10]. By contrast, endorepellin, the C-terminal fragment of perlecan, is a powerful inhibitor of angiogenesis by inducing actin disassembly and focal adhesion disruption in endothelial cells [6]. The anti-angiogenic activity of endorepellin is based on its binding to integrin α2β1, followed by activation of the phosphatase SHP-1 (Src homology 2 domain phosphatase-1), which then dephosphorylates, thus inactivating receptor tyrosine kinases including vascular endothelial growth factor (VEGF) receptors [11].

#### Elastin

Together with other large glycoproteins, such as, fibrillin and fibulin, elastin is a component of the elastic tissue. It is present in the dermis and several other connective tissues such as vessel walls. Several diseases are linked to mutations in components of the elastic tissue, including some forms of cutis laxa or Marfan syndrome [12]. Elastic fibers are not detected in the granulation tissue synthesized during early phases of wound healing, indicating that a certain differentiated status of fibroblasts is required to produce elastic tissue [13].

#### Proteoglycans

Proteoglycans are composed of positively charged polysaccharide chains attached to a protein backbone. They can be very large and are hydrophilic, which endows them with high water-binding capacity, and confers viscoelasticity and compression resistance to tissues. Proteoglycans are found in basement membranes, on cells and in matrices surrounding mesenchymal cells. Perlecan, a heparan sulfate proteoglycan, has (in addition to its structural role) a number of signaling functions via its heparan sulfate chains, which bind and modulate growth factors such as fibroblast growth factor (FGF)2, VEGF and platelet-derived growth factor [14]. The importance of heparan sulfate chains in growth factor regulation has been shown in perlecan heparan sulfate deficient-mice, which display delayed wound healing and poor vascularization of wound tissue due to reduced FGF2-mediated angiogenesis [15]. Further, overexpression of recombinant heparanase improves wound healing by increasing mature α-smooth muscle actin-positive blood vessels and by accelerating wound closure and epithelialization. The proposed mechanism is mobilization of heparan-bound growth factors from the ECM [16]. Interestingly, degradation products of hyaluronan, which is a large, unsulfated glycosaminoglycan without an associated protein core, led to an increase in the number of blood vessels [17].

#### Fibronectin

One of the best-characterized glycoproteins of the ECM is fibronectin, a major product of dermal fibroblasts and myofibroblasts, which has a wide distribution in the dermis, in the dermal-epidermal basement membrane zone. Fibronectin is a large adhesive glycoprotein with a huge number of different functions. Besides its structural role, fibronectin is the main cell adhesion molecule, modulating many cellular activities. In humans, alternative splicing generates close to 20 fibronectin variants with different functions, of which the ED-A variant is an important regulator of fibroblast to myofibroblast differentiation [18]. After injury, fibronectin is initially deposited from blood plasma, and plays an important role in platelet function with the release of growth factors and cytokines [19]. Together with fibrin it provides most of the provisional matrix in dermal wounds, guiding fibroblasts and inflammatory cells to the site of injury. In restoring a neodermis, fibroblasts secrete fibronectin dimers, which are organized into a dense pericellular network in a cell-mediated and integrin-dependent fashion [20]. The formation of a stable collagen I/III fibrillar network is thought to depend on a pre-existing fibronectin network through a mechanism involving integrins α2β1 and α11β1 [21,22].

#### Laminins

Laminins represent a protein family of 16 currently known members. Laminins are large trimeric ECM glycoproteins consisting of three different polypeptide chains [23]. Laminin chains self-polymerize and interact with other basement membrane proteins such as collagen IV, perlecan and nidogen. They are the major constituents of the dermoepidermal basement membrane and that of blood vessels. Laminins control cell migration, differentiation and proliferation. In skin repair, laminins are key molecules regulating re-epithelialization and vascularization. Laminins 411 and 511 are the major isofoms in the basement membranes of blood vessels. They are produced by human dermal microvascular endothelial cells and pericytes [24].

#### Matricellular proteins

More recently a group of proteins named the matricellular proteins was identified. These proteins interact with other structural ECM molecules and cell surface receptors, and modulate cytokine activation. They are thought to function as important regulatory molecules rather than as structural components [25].

The multi-domain protein thrombospondin represents a family of five members, of which thrombospondin-2 is the predominant member; it is expressed by fibroblasts and endothelial cells in murine skin. Thrombospondin-1 is found in platelets and is secreted by inflammatory cells [26,27]. Thrombospondin-1 and -2 have been ascribed anti-angiogenic activities [28]. Both forms are specifically expressed at different phases during tissue repair, thrombospondin-1 in the early and thrombospondin-2 in the late phases. In early wound stages, thrombospondin-1 act as a chemoattractant for macrophages [29], and modulates transforming growth factor (TGF)-β activation and signaling. As a likely result, wounds in thrombospondin-1 null mice heal at lower rates. In mice overexpressing thrombospondin-1 in skin, wound healing is delayed, and associated with diminished angiogenesis and reduced granulation tissue formation [30]. In thrombospondin-2 null mice, wound healing is accelerated and the microvascular network is increased [27]. These observations indicate that the functions of thrombospondin-1 and -2 in skin are dictated primarily by their spatiotemporal expression patterns. Less information is available on the role of the other thrombospondin family members. However, despite distinct, non-redundant roles in specific tissues, they all support cell attachment and binding to other ECM proteins [31].

The secreted acidic and cysteine-rich protein family includes SPARC (secreted protein acidic and rich in cysteine, also known as osteonectin and BM40) and hevin, both expressed in skin, primarily by fibroblasts [32]. *In vitro*, both proteins exhibit de-adhesive and anti-migratory activities. Wound closure is accelerated in both SPARC and hevin null mice [33,34]. However, in hevin null mice, accelerated wound closure was in part attributed to its de-adhesive and anti-migratory function for dermal fibroblasts [34], whereas in SPARC null mice, acceleration was attributed to decreased collagen content rendering the skin more susceptible to cell contraction [33].

The matricellular family of tenascin proteins comprises four members in mammals. Tenascins are large glycoproteins with structural similarity to fibronectin. Like SPARC and thrombospondin-1, they do not support cell adhesion. Tenascin-C is abundantly present in the provisional wound matrix, and its synthesis by fibroblasts is strongly enhanced by mechanical tension [35]. Skin wounds of tenascin-C null mice display diminished fibronectin but close normally [36]. Tenascin-X is far less abundant than tenascin-C in skin. Patients deficient in tenascin-X have a rare variant of Ehlers-Danlos syndrome, with skin hyperextensibility [37]. Although patient skin is characterized by reduced density of collagen fibers and abnormal elastin fibers [38], scars are not atrophic but tend to be wider than normal, and scar tissue in tenascin-X null mice has reduced stability [39].

### The extracellular matrix and growth factors

Many ECM constituents actively modify the activity of growth factors and cytokines and some act as a reservoir from which growth factors can be rapidly released if required (Table 1). The ECM can also protect growth factors from degradation [40,41].

**Table 1:** Growth factors binding to the extracellular matrix

basic fibroblast growth factor (FGF-2)	heparan sulfate proteoglycan
transforming growth factor β (TGF-β)	decorin, thrombospondin-1
vascular endothelial growth factor (VEGF)	heparan sulfate proteoglycan
platelet derived growth factor (PDGF)	heparan sulfate proteoglycan

Probably the best example is the binding of FGF-2 to the heparan sulfate proteoglycan, perlecan. This binding promotes FGF receptor activation and ultimately downstream signaling, which supports mitogenesis and angiogenesis [42]. Other examples are the inactivation of TGF-β by binding to decorin [43], and the activation of TGF-β by binding to thrombospondin-1 [44]. VEGF also binds to heparin or proteoglycan binding domains within various ECM molecules, and release of VEGF by plasmin or heparanase triggers endothelial cell proliferation [45]. Further enhanced endothelial cell proliferation is achieved by VEGF binding to the heparin II domain in fibronectin if the same fibronectin molecule is bound to the endothelial cell via integrin α5β1. This example highlights a novel mechanism implicating the ECM in bringing a signaling integrin receptor and a growth factor receptor in close apposition to facilitate angiogenesis [46].

These modifications in the availability and activity of growth factors and cytokines by defined constituents of the ECM contribute to the versatile response of cells to a changing environment. This interaction is most obvious in situations in which large amounts of active cytokines are required, such as in wound healing and tissue remodeling. However, ECM modulation is equally important in situations when cellular activities must be turned down to reduce tissue damage and scarring, as in late stages of tissue repair or after inflammatory processes.

Within the past few years, a new class of ligands, the matrikines and matricryptins, has been characterized as subdomains of various ECM proteins, which can signal to cells through receptors such as growth factor receptors [47]. The EGF-like repeats in tenascin-C and laminin are examples of matrikines. At these sites they bind to EGF receptors, thereby enhancing cell motility [47,48]. In the case of laminin 332, this activity appears to occur only after cleavage by matrix metalloproteinases (MMPs) [49]. During wound healing, tenascin-C and laminin 332 are expressed by keratinocytes at the leading edge, correlating temporally with keratinocyte migration and MMP-2 expression. Based on this, binding of the released laminin fragment to the epidermal growth factor receptor (EGFR) is believed to provide pro-migratory tracks within the edges of the healing wound for migrating and proliferating keratinocytes, whereas binding of tenascin-C may enhance migration and de-adhesion of cells [4,50]. Decorin, which acts as a suppressor of cellular proliferation, is another EGFR binding matrikine. Binding results in decreased EGFR levels and suppression of EGFR autophosphorylation [51]. Hence, during wound repair, matrikine signaling from decorin could function as a quiescence signal eliciting decreased migration, proliferation and ECM synthesis by fibroblasts after the wound has reached a mature state [50].

In summary, the individual domains of the ECM can interfere with growth factor signaling either by direct interaction with growth factor receptors or indirectly by controlling the availability and activity of growth factors and cytokines. This versatility of ECM components in regulating cellular activities is further expanded by close cooperation between growth factor signaling and integrin-mediated cell-matrix interactions and mechanical tension (discussed in detail below).

## Integrins and cell-matrix interactions

Although many receptors for ECM proteins have been identified, the integrin family probably constitutes the most abundant receptors mediating the interaction of cells with their surrounding ECM. Integrins have a relatively restricted, cell type-specific and context-dependent expression with defined substrate specificity. Ligands bind to the extracellular domains of integrins, and integrin cytoplasmic tails connect via linker proteins to the cytoskeleton. Integrins mediate cell adhesion, migration and survival, and also specific differentiation programs relevant to development, tissue maintenance and repair. By virtue of linking the outside environment with the cellular inside, they transduce signals and mechanical tension bidirectionally from the outside in and the inside out [52,53].

**Figure 1:**
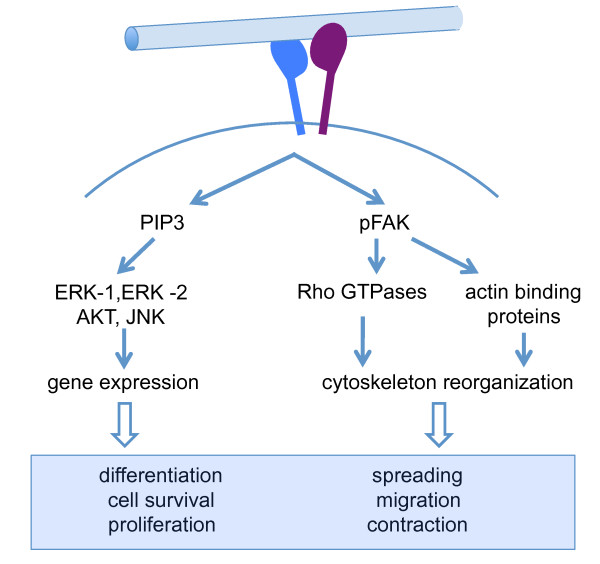
**Integrin signaling**. Activated integrins bind to extracellular matirix (ECM)ligands and signal via phosphorylation of intracellular linker proteins or lipid second messengers and small GTPases, eliciting reorganization of the cytoskeleton, changes in gene expression and modulation of many cellular responses. AKT, protein kinase B; ERK, extracellular signal-regulated kinase; JNK, Jun N-terminal kinase, PIP3, phosphatidyl inositol triphosphate.

Integrin function is closely connected with integrin conformation [54]. Usually, integrins are expressed at the cell surface in an inactive form. Signals arising from the cytoskeleton (inside-out signaling) modulate integrin conformation. Talin and kindlin were recently described as key linker proteins responsible for integrin activation [55,56]. Activation results in increased affinity for ECM molecules and clustering into focal adhesions [57,58] with rapid tyrosine phosphorylation of specific substrates and an increase in the concentration of lipid second messengers. This is then followed by cytoskeletal rearrangements, modulation of Rho GTPase activity, and reorganization of actin binding proteins, affecting virtually all cellular functions [59] (Figure 1).

Integrin expression and activity can be further modulated by other transmembrane proteins such as tetraspanins or cell surface heparan sulfate proteoglycans. Most of these are expressed in a cell type-specific manner in different phases of skin repair and scarring [60].

Activation of integrins, transmission of mechanical tension originating from tissue remodeling, and the regulated activity of growth factors play crucial roles in controlling cellular activities during dermal repair, scarring and fibrosis. Activation of TGF-β by αv integrins is a good example of such interplay. Binding of latent TGF-β to integrin αvβ6, which is abundantly expressed by keratinocytes in wound epithelium and in chronic wounds [61], induces a conformational change in the latent complex, resulting in unmasking of the mature TGF-β moiety, presentation to TGF-β receptors on adjacent cells, and induction of TGF-β signaling in a strictly locally confined manner [62,63]. By contrast, integrin αγβ8 binding to the same site in the latent TGF-β complex is independent of cell-cell contact and instead presents the latent complex to membrane-bound MMPs that reside in close vicinity to the integrin in the same cell. Proteolytic activation of the latent complex releases freely diffusible mature TGF-β that is active at distant sites [64].

Angiogenesis can be controlled by the crosstalk between αvβ3 and FGF that results in modulation of FGF signaling [65,66], and by the stable interaction between integrin α5β1 and Tie-2 tyrosine kinase receptor that regulates endothelial cell response to angiopoietin-1 [67].

The mechanisms by which integrins modify cellular responses to growth factors can involve direct regulation of transcriptional programs, complex activation mechanisms or various signaling cascades. The data available also clearly demonstrate a close link between the ECM, the cytoskeleton and growth factor/cytokine release, and the cellular response. Obviously, most experiments have been based on monolayer culture systems, and only limited information is available on how cells interact with the ECM in a complex three-dimensional system such as the dermis. In those situations, the cellular architecture is modified and mechanical tension is generated, which modulates integrin functions and the cellular response to growth factors.

## Regulation of fibroblast activation

Although there is increasing evidence that in certain specialized tissues, epithelial cells participate in fibrosis and scarring, in most tissues fibroblasts are the main effector cells for the production and deposition of new and sometimes excessive ECM. Fibroblasts were among the first cells grown in culture dishes, but specific markers are still lacking and their differentiation is only incompletely understood. They are generally defined by their morphology and by a characteristic pattern of gene expression. In tissue repair, they synthesize most ECM molecules and produce proteases required for the remodeling in later phases. Fibroblasts also secrete numerous growth factors and cytokines that act on other cell types thereby modulating tissue repair and scarring [68,69].

After tissue damage and the initial inflammatory reaction, fibroblasts are modulated from quiescent cells to migratory, proliferating and biosynthetically active cells. It is well established that they can be transformed into myofibroblasts, the contractile activated cell type, which is mainly responsible for scar contraction and excessive ECM deposition [68]. The finely tuned balance between the activated and quiescent fibroblast subpopulations seems to determine scar quality.

### Origin of fibroblasts in wounds, scars and fibrosis

The exact origin of fibroblasts in wounds or fibrotic tissue remains unclear and is still a matter of debate. A certain fraction of fibroblasts within wounds and scars originates from resident cell populations. In normal wounds, the balance between growth factor activity and the integration of fibroblasts into an environment with stable mechanical tension ensures cell survival and gene expression patterns required for normal tissue remodeling [70]. Changes in mechanical stiffness and in release of growth factors from inflammatory cells can be the initial stimulus for the activation of resident tissue fibroblasts, with formation of contractile actin stress fibers and firm attachment via fibroblast integrins to the ECM [71,72].

Many studies have considered alternative origins of fibroblasts in scarring and fibrosis. A significant percentage of fibroblasts in dermal wounds was derived from bone marrow in green fluorescent protein transgenic mice [73]. There is also evidence that bone marrow-derived cells contribute to the disease process in certain fibrotic diseases [74]. However, it is not clear whether or how these cells are related to circulating fibrocytes, the leukocyte subpopulation characterized by collagen production and surface markers indicative of hematopoietic origin, which have been described to contribute to wound healing and fibrosis [75,76].

**Figure 2:**
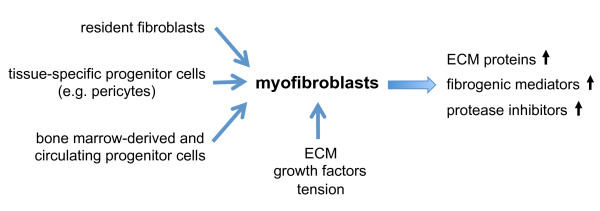
**Formation of myofibroblasts and their effects on extracellular matrix (ECM) homeostasis**. Myofibroblasts may arise from various cellular sources. Their differentiation and activity are modulated by the surrounding ECM, growth factor activity and mechanical tension. Myofibroblasts affect connective tissue homeostasis by upregulated production of ECM proteins, fibrogenic mediators and protease inhibitors.

Although this field is rapidly developing, the specific roles of these different fibroblast progenitor populations are incompletely understood. It is likely that the extent of matrix deposition and tissue destruction in wounds or inflamed tissue depends on the local environment, ECM composition, magnitude of integrin-mediated mechanical stress, and growth factors released by different populations of circulating progenitors (Figure 2).

### Myofibroblast formation

Myofibroblasts have long been associated with wound repair and fibrosis [77]. They are contractile cells, which are thought to develop from fibroblasts after specific external stimuli or from circulating mesenchymal precursor cells (see previous section). The prime function of myofibroblasts is reconstruction of granulation tissue by producing new ECM, and contraction and remodeling of wound and scar tissue. Myofibroblasts are key effectors in hypertrophic scars and have recently also been implicated in fibrotic processes, for example, in scleroderma.

Previously, myofibroblasts were characterized ultrastructurally by electron microscopy. They differ from normal fibroblasts by the expression of α-SMA, which is the most widely used marker for myofibroblasts. They are also characterized by expression of the ED-A splice variant of fibronectin and the upregulated synthesis of several ECM proteins and growth factors [78]. Induction of ED-A fibronectin, in turn, requires presence of TGF-β together with mechanical tension, indicating that complex paracrine and autocrine networks are involved in regulating myofibroblast formation in wound healing [70,78]. In addition, keratinocyte-fibroblast interactions in coculture systems have been reported to lead to myofibroblast formation. In this system, TGF-β together with endothelin-1 have been identified as responsible factors [79].

Myofibroblasts promote wound and scar contraction by establishing cell-cell and cell- ECM contacts and tractional forces. They form bundles of contractile microfilaments and extensive cell matrix contacts, which attach the microfilaments to the surrounding ECM. These contacts (the so-called fibronexi) have been defined ultrastructurally [80]. Formation of supermature focal adhesion structures and establishment of the α-SMA-rich network of stress fibers are hallmarks of myofibroblasts. They provide an integrin-based system for the transduction of mechanical forces between these cells and the surrounding ECM [72,81].

In the later stages of normal wound repair, myofibroblasts undergo apoptosis and disappear. Loss of mechanical tension, alterations in cell density or protease activity and/or changes in the pattern of secreted growth factors are thought to contribute to cell death. Understanding the exact cellular and molecular basis for clearance of myofibroblasts is essential because in pathological situations (e.g. scarring or fibrosis), myofibroblasts persist and are responsible for some of the clinical symptoms.

### Other cell types involved in ECM deposition in scar formation and fibrosis

Many other cell types either directly or indirectly contribute to scar formation. The early inflammatory infiltrate is initially activating a complex cascade of factors, which depend on one another and profoundly influence late events after most of the inflammatory cells have disappeared.

In certain tissues, epithelial-mesenchymal transition plays an important role during development, but has more recently also attracted attention for explaining the development of fibrosis, for example, in kidney and possibly in lung. In response to growth factors and altered ECM contacts, epithelial cells can lose their epithelial polarity and adopt a mesenchymal phenotype [69].

The microvascular pericytes are integral parts of all microvessels, and play an important role in regulating endothelial cells. Although the precise function of pericytes is not clear they crucially contribute to stabilize and mediate forces within the microvasculature [82]. Pericytes contain contractile proteins and can probably generate contractile forces. In fibrosis and dermal repair, pericytes become activated, and may represent a pool of mesenchymal precursor cells in the tissue.

### The role of mechanical tension

All adherent cells including endothelial cells, fibroblasts and myofibroblasts sense tension originating from the environment. Tension is transmitted via cell-ECM contacts [83], leading to reorganization of the cytoskeleton and the elicitation of specific signals that modulate gene expression. In muscles, bone, tendon, skin and vessels, alterations of mechanical forces are continuously recognized by cells and their functions are adapted according to the biological requirements. If mechanical tension is removed, those tissues undergo atrophy, indicating the important role of mechanical signals for maintaining proper functioning of the organism [84].

Obviously, fibroblasts and myofibroblasts are cells implicated in scarring, which is strongly influenced by mechanical tension. Both cell types firmly attach to ECM structures via matrix adhesions, which have been studied in detail in monolayer cultures (see previous sections). These include the focal complexes, focal adhesions and fibrillar adhesions. The major structures required to form such matrix contacts are the integrin receptors, which directly connect the ECM structures to the intracellular cytoskeleton network [85]. Fibroblasts and myofibroblasts in the skin use α5β1 or αv integrins to bind to fibronectin, whereas collagens are primarily recognized by integrins α1β1, α2β1 and α11β1 and to a lesser extent by other receptors such as cell surface heparan sulfate proteoglycans (syndecans) or discoidin domain receptors (Table 2). Mechanical forces act on focal adhesions, resulting in further structural maturation [72,86].

**Table 2:** Collagen receptors

*Integrin receptors recognizing fibrillar collagens*	
collagen I	α1β1, α2β1, α11β1
collagen II	α10β1
collagen III	α1β1, α2β1, α11β1
collagen V	α1β1, α2β1
collagen I fibrils	α2β1, α11β1
denatured collagen I	αvβ
*Non integrin collagen receptors*	
discoidin domain receptor-1 and -2 (DDR1,DDR2)	
glycoprotein VI (GPVI)	
leukocyte-associated immunoglobulin-like (LIAR-1)	
syndecans	
glypicans	
CD44	

More recently, fibroblasts have been grown in three-dimensional ECM structures, in which cell shape is modified and they become bipolar. A clear distinction of the different attachment sites is more difficult, but these structures are equally important and establish a direct connection between the surrounding ECM and the cell nucleus [87,88]. Using collagens as three-dimensional structures, several groups have demonstrated that applying tension to the matrix directly affects the biosynthetic capacities of fibroblasts [89].

## Transmission of signals

The precise mechanisms by which different cell types transmit mechanical signals are not fully understood. They might involve stretch-activated ion channels, direct interactions between structural and signaling components, or activation of small GTPases [90].

As outlined above, many cooperative interactions exist between integrins and growth factor signaling. In particular, fibroblast to myofibroblast conversion and α-SMA expression crucially depend on a combination of mechanical tension and TGF-β [81]. Thus in scarring, generation of tension can induce myofibroblast formation, causing a self-perpetuating loop. A similar autocrine loop is discussed for the induction of collagen synthesis in fibroblasts by mechanical tension. In this case, TGF-β is induced by tension, which in turn activates collagen synthesis via the classic pathways [91]. In addition, fibronectin is induced by the application of cyclic strain to fibroblasts. In parallel, many proteases are downregulated, whereas protease inhibitors are upregulated. Extensive work has concentrated on tenascin-C, which is found in tissues with high tensile stress [35]. Detailed analysis using several *in vitro *systems has demonstrated that tenascin-C expression is strongly upregulated at the transcriptional level by mechanical stress, involving specific *cis*-acting elements in the tenascin-C promoter [92,93].

These data clearly demonstrate that mechanical tension, which is generated during wound contraction, scar formation and in fibrotic tissue, can modulate the gene expression of fibroblasts and myofibroblasts embedded into this tissue at different molecular levels. Tension directly modifies gene transcription, induces signaling from integrins affecting small GTPases or induces/inhibits growth factor signaling, which then indirectly affects ECM protein synthesis by fibroblasts/myofibroblasts. By a combination of these mechanisms, mechanical tension induces an activated, contractile fibroblast/myofibroblast phenotype characterized by high levels of synthesis of ECM proteins, low protease activity and high production of fibrogenic cytokines.

## Conclusions and perspectives

**Figure 3:**
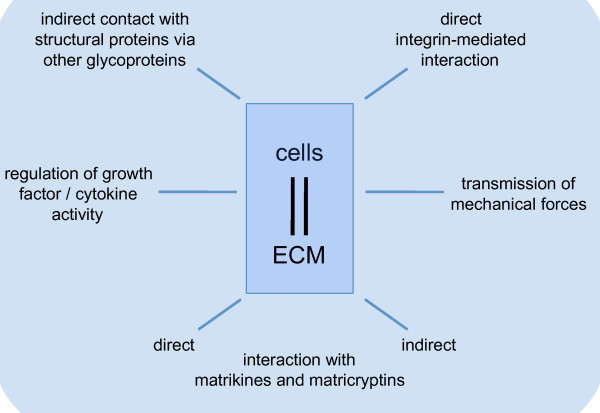
**Interaction of cells with the extracellular matrix (ECM) during dermal repair**. This schematic illustrates different types of cell-ECM interactions. For details, please refer to the text.

Interaction of cells with the surrounding ECM is an important modulator of all cellular activities (Figure 3). Tight control is required, particularly to ensure homeostatic conditions during development, tissue repair and in fibrotic situations, in which cellular activities are either turned on or downregulated. Crosstalk with the surrounding ECM is essential for the function of all cells, and in the context of scarring and fibrosis it is probably most crucial for fibroblasts and myofibroblasts.

The interaction of these cells with different ECM constituents is mediated by several integrins that signal bidirectionally and transmit mechanical forces that are generated during pathological situations. Cell-ECM interactions mediated by integrins are closely linked to growth factor signaling and to complex modulation of the cellular cytoskeleton. This complex interplay controls the specific gene expression program at any given time. Detailed insight into the roles of different ECM proteins, the function of integrins, their modulation by forces, and their interplay with growth factors will help us to understand the orchestration of tissue architecture and the processes underlying excessive scarring and fibrosis.

## Competing interests

The authors declare that they have no competing interests.

## Authors' contributions

BE, RN and TK drafted the manuscript. TK was responsible for its design and coordination. All authors read and approved the final manuscript.

## Appendices

**Appendix 1: **Composition of the extracellular matrix in skin

• ***Structural proteins: ***collagens, elastin, fibrillins, fibulins

• ***Adhesive glycoproteins: ***fibronectin, vitronectin, matrilins, laminins

• ***Proteoglycans: ***perlecan, syndecans, versican, decorin, biglycan, lumican

• ***Matricellular proteins: ***thrombospondins, tenascins, SPARC/BM-40/osteonectin

Appendix 2: The collagen subfamilies

• Fibril-forming collagens (I, II, III)

• Fibril-associated collagens with interrupted helices (IX, XII, XIV, XX, XXI, XVI)

• Hexagonal network-forming collagens (VIII)

• Basement membrane-associated collagens (IV, VII, XVII, XVIII)

• Beaded-filament forming collagens (VI)

• Transmembrane collagens (XIII, XVII, XXIII, XXV)

• Multiplexins (XV, XVIII)
